# Knowledge, attitudes, and practices related to pulmonary rehabilitation and associated influencing factors among older adults with chronic obstructive pulmonary disease in a county medical community

**DOI:** 10.3389/fpubh.2026.1781124

**Published:** 2026-03-16

**Authors:** Ting-Yan Li, Yan-Hua Hu, Jian Wang, Wen-Yan Hu

**Affiliations:** 1 Department of Respiratory Medicine, Linping Campus, Second Affiliated Hospital, Zhejiang University School of Medicine, Hangzhou, China; 2 Department of General Practice, Linping Campus, Second Affiliated Hospital, Zhejiang University School of Medicine, Hangzhou, China

**Keywords:** chronic obstructive pulmonary disease, county medical community, influencing factors, knowledge-attitude-practice, pulmonary rehabilitation

## Abstract

**Background:**

Chronic obstructive pulmonary disease (COPD) poses a significant global health burden, particularly in older adults. Pulmonary rehabilitation (PR) is a core management strategy, yet its implementation remains limited and imbalanced, especially within primary healthcare settings in China. The county medical community model aims to integrate healthcare resources, representing a potential platform for optimizing PR delivery. This study aimed to assess the knowledge, attitudes, and practices (KAP) regarding PR among older adults with COPD within a county medical community and to analyze the associated influencing factors.

**Methods:**

A convenience sampling approach was applied to select 150 older adults with COPD who visited medical community institutions between January 2024 and December 2024. Data were collected using a general information wquestionnaire and a pulmonary rehabilitation KAP questionnaire. Statistical analyses were conducted to evaluate KAP levels and to identify factors associated with pulmonary rehabilitation KAP.

**Results:**

The total pulmonary rehabilitation KAP score among the 150 older adults with COPD was 68.63 ± 19.23. Scores for the knowledge, attitude, and practice dimensions were 32.57 ± 11.79 (out of a possible 60), 15.77 ± 3.77 (out of a possible 20), and 20.28 ± 8.50 (out of a possible 50), respectively. Multiple linear regression analysis indicated that the type of hospital visited, age, the presence of a primary caregiver, and guidance from medical staff were significant influencing factors associated with pulmonary rehabilitation KAP among older adults with COPD (*p* < 0.05).

**Conclusion:**

Older adults with COPD in the medical community demonstrated a generally positive attitude toward pulmonary rehabilitation, whereas knowledge and practice levels remained relatively low. Strengthened and targeted professional guidance from medical staff for patients and their caregivers across different age groups is warranted. Enhanced coordination within medical communities and the development of innovative service models may facilitate more effective implementation of pulmonary rehabilitation, thereby improving quality of life and health management among older adults with COPD.

## 1 Introduction

Chronic obstructive pulmonary disease (COPD) is a major contributor to global morbidity and mortality, with a particularly high and increasing burden among older adults, and it substantially compromises quality of life among affected patients (1, 2). Pulmonary rehabilitation (PR), as a core component of COPD management, has been demonstrated to improve exercise capacity, alleviate symptoms, and enhance quality of life in patients with COPD (3). Primary healthcare institutions serve as the frontline settings for COPD prevention, treatment, and long-term management and therefore play a critical role in the delivery of PR care (4). However, the implementation of PR across medical institutions at different levels in China remains limited and imbalanced, particularly in county-level and primary healthcare institutions, where the development and dissemination of PR continues to encounter substantial barriers (1, 5).

The County Medical Community, hereafter referred to as the medical community, has emerged in recent years as a key strategy to advance healthcare system reform in China. This model is characterized by integrated county and township management, with county hospitals assuming a leading role, township health centers functioning as central hubs, and village clinics serving as the foundational units. Through this structure, county hospitals can more effectively bridge urban and rural healthcare resources and promote the prevention, treatment, and management of chronic diseases at the primary care level (6). Existing evidence indicates that patients’ knowledge of and attitudes toward PR are closely associated with their willingness to participate in and adhere to PR programs (7). Accordingly, a comprehensive understanding of Knowledge–Attitude–Practice (KAP) related to PR among patients with COPD within the medical community is of particular importance for optimizing COPD management strategies.

Based on this context, the present investigation assessed the current status of PR-related KAP among older adults with COPD within the medical community model, with the aim of providing targeted evidence to support the effective promotion and continuous optimization of PR services in medical communities.

## 2 Data and methods

### 2.1 Study participants

This study was conducted within a single county medical community in Zhejiang Province, China. The medical community consists of one tertiary hospital (the lead unit) and three affiliated primary healthcare institutions (township health centers) operating under the integrated county medical community model. All four institutions are located within the same administrative region (the same county). A random sampling survey was conducted among older adults with COPD who visited these institutions within the medical community between January 2024 and December 2024. The inclusion criteria were as follows: (1) patients who met the diagnostic criteria outlined in the *Guidelines for the Diagnosis and Treatment of Chronic Obstructive Pulmonary Disease (2021 Edition)* formulated by the Chinese Thoracic Society and who had received a confirmed clinical diagnosis of COPD; (2) age ≥ 60 years; and (3) willingness to participate in the survey. The exclusion criteria included the following: (1) coexistence of tumors, mental disorders, or other conditions that significantly affect motor function; and (2) inability to complete the questionnaire or poor cooperation during data collection. Questionnaires containing invalid responses or missing items were excluded from the analysis. Ethical approval for this investigation was obtained from the Ethics Committee of the Linping Campus, Second Affiliated Hospital, Zhejiang University School of Medicine (Approval no. 2023–135). Written informed consent was obtained from all participants.

### 2.2 Methods

#### 2.2.1 Survey tools

Two survey instruments were used. (1) A general information questionnaire was developed based on a literature review and included variables such as the medical institution visited, sex, age, education level, marital status, presence of a primary caregiver, type of medical insurance, smoking history, disease duration, number of hospitalizations due to acute exacerbations in the past year, presence of comorbidities, and PR training provided under the guidance of medical staff. (2) The KAP questionnaire on PR training for patients with COPD, developed by Huo Shaojuan, was applied (8). The overall Cronbach’s *α* coefficient of the questionnaire was 0.852. Cronbach’s α coefficients for the knowledge, attitude, and practice dimensions were 0.743, 0.909, and 0.875, respectively, indicating satisfactory reliability and validity. The questionnaire consisted of 12 items assessing PR-related knowledge (total possible score: 60), four items assessing attitudes (total possible score: 20), and 10 items assessing practices (total possible score: 50). All items were rated using a 5-point Likert scale. The knowledge dimension was scored as 1–5 points corresponding to “very unclear,” “unclear,” “generally clear,” “clear,” and “very clear,” with higher scores indicating greater knowledge of PR. The attitude dimension was scored as 5–1 points, corresponding to “strongly agree,” “agree,” “general,” “disagree,” and “strongly disagree,” respectively, with higher scores reflecting more positive attitudes. The practice dimension was scored as 1–5 points corresponding to “never,” “occasional,” “sometimes,” “often,” and “always,” with higher scores indicating better practice levels.

#### 2.2.2 Data collection methods

Investigators involved in data collection completed standardized training on survey procedures and methods prior to implementation. Following successful completion of the training, on-site investigators explained the study purpose and questionnaire requirements to eligible participants. After informed consent was obtained, participants completed the paper-based questionnaires independently or, when necessary, responses were recorded by investigators based on patient descriptions. Data collection followed principles of voluntary participation as well as anonymity and confidentiality. A total of 150 questionnaires were distributed, and all 150 were deemed valid, yielding an effective response rate of 100%.

#### 2.2.3 Statistical methods

The sample size was estimated based on Kendall’s sample size estimation method for multivariate analysis. Given that the KAP questionnaire comprises 26 items, a minimum of 5–10 participants per item is recommended. The minimum required sample size was calculated as 26*5 = 130 participants. To compensate for an anticipated 10% rate of invalid or non-response questionnaires, the sample size was increased to 130 / (1–0.10) ≈ 145 participants. Ultimately, 150 participants were enrolled to ensure the robustness of the analysis.

Statistical analyses were performed using SPSS version 26.0. Categorical variables were described using frequencies and percentages. Continuous variables that conformed or approximately conformed to a normal distribution, as assessed by normality testing, were described using mean ± standard deviation. Between-group comparisons were conducted using independent-samples t tests or analysis of variance. Multiple linear regression analysis was applied to examine factors associated with KAP related to PR among older adults with COPD. A two-sided *p* < 0.05 was considered statistically significant.

## 3 Results

### 3.1 KAP scores of PR in older adults with COPD

Among the 150 participants with COPD, the total KAP score related to PR was 68.63 ± 19.23. The mean scores for the knowledge, attitude, and practice dimensions were 32.57 ± 11.79 (out of a possible 60), 15.77 ± 3.77 (out of a possible 20), and 20.28 ± 8.50 (out of a possible 50), respectively. The score rates for the knowledge, attitude, and practice dimensions, as well as the overall KAP score, were calculated using the formula: average score divided by the theoretical maximum score multiplied by 100%. The mean scores and corresponding score rates for each dimension are presented in Table 1.

**Table 1:** KAP scores related to pulmonary rehabilitation among older adults with COPD (points, $\bar{\chi }\pm s$ ).

Item	Theoretical minimum	Theoretical maximum	Scores	Scoring rate (%)
Scores of knowledge	12	60	32.57 ± 11.79	54.29
Scores of attitude	4	20	15.77 ± 3.77	78.87
Scores of practice	10	50	20.28 ± 8.50	40.56
KAP total scores	26	130	68.63 ± 19.23	52.79

### 3.2 Univariate analysis of factors associated with KAP related to PR

The results of the univariate analysis of factors associated with PR-related KAP among older adults with COPD in the medical community are presented in Table 2. Statistically significant differences in KAP scores were observed according to the type of medical institution visited, age group, presence of a primary caregiver, number of hospitalizations due to acute exacerbations in the past year, and receipt of professional PR guidance from medical staff (*p* < 0.05).

**Table 2:** Univariate analysis of factors associated with KAP related to pulmonary rehabilitation among older adults with COPD (points, $\bar{\chi }\pm s$ ).

Item	*n* (%)	KAP score	*t/F-*value	*p-*value
Sex			0.36	0.723
Male	132 (88.00)	68.83 ± 19.11		
Female	18 (12.00)	67.11 ± 20.60		
Hospital visited			2.38	0.019
County-level hospital	53 (35.33)	73.60 ± 20.68		
Primary healthcare institution	97 (64.67)	65.91 ± 17.93		
Age			3.45	0.010
60–65 years old	34 (22.67)	76.76 ± 19.37		
66–70 years old	36 (24.00)	69.64 ± 21.25		
71–75 years old	40 (26.67)	68.28 ± 18.85		
76–80 years old	26 (17.33)	63.04 ± 14.93		
> 80 years old	14 (9.33)	57.64 ± 14.44		
Education level			1.87	0.120
Illiterate	15 (10.00)	56.47 ± 19.62		
Primary school	89 (59.33)	69.12 ± 17.97		
Junior high school	35 (23.33)	71.66 ± 20.49		
High school	5 (3.33)	69.40 ± 23.31		
University and above	6 (4.00)	73.33 ± 20.83		
Marital status			1.92	0.150
Married	127 (84.67)	69.80 ± 18.76		
Divorced	6 (4.00)	68.00 ± 16.17		
Widowed	17 (11.33)	60.12 ± 22.45		
Is there a primary caregiver			−3.18	0.002
No	104 (69.33)	64.82 ± 14.98		
Yes	46 (30.67)	77.24 ± 24.53		
Types of medical insurance coverage for healthcare			2.48	0.064
Urban and Rural Residents Medical Insurance	114 (76.00)	66.47 ± 19.06		
Employee Medical Insurance	30 (20.00)	74.23 ± 17.12		
Self-paid	3 (2.00)	75.67 ± 21.50		
Other Medical Insurances	3 (2.00)	87.33 ± 31.47		
Smoking history			−0.43	0.671
No	33 (22.00)	67.36 ± 20.12		
Yes	117 (78.00)	68.98 ± 19.05		
Disease course			0.25	0.776
≤ 2 years	43 (28.67)	66.98 ± 20.05		
2–10 years	51 (34.00)	69.80 ± 18.67		
> 10 years	56 (37.33)	68.82 ± 19.36		
Number of hospitalizations due to acute exacerbation in the past year			4.85	0.009
0	40 (26.67)	65.33 ± 18.00		
1	79 (52.67)	66.66 ± 20.04		
≥ 2	31 (20.67)	77.90 ± 16.05		
Presence of other comorbidities			1.19	0.237
No	35 (23.33)	72.00 ± 19.32		
Yes	115 (76.67)	67.60 ± 19.17		
Received PR training under the guidance of medical staff			−6.12	<0.001
No	93 (62.00)	61.88 ± 17.24		
Yes	57 (38.00)	79.63 ± 17.24		

### 3.3 Multiple linear regression analysis of factors associated with KAP related to pulmonary rehabilitation

The total PR-related KAP score was used as the dependent variable. Independent variables included factors that demonstrated statistically significant associations in the univariate analysis. The coding and assignment of independent variables are presented in Table 3. Multiple linear regression analysis indicated that the type of medical institution visited, age, presence of a primary caregiver, and receipt of guidance from medical staff were independently associated with PR-related KAP among older adults with COPD (*p* < 0.05), as presented in Table 4.

**Table 3:** Coding and assignment of independent variables included in the multiple linear regression analysis.

Independent variables	Assignment		
Hospital visited	County-level hospital = 1	Primary healthcare institution = 2	
Age	60–65 years old = 1	66–70 years old = 2	71–75 years old = 3
	76–80 years old = 4	>80 years old = 5	
Is there a primary caregiver	No = 0	Yes = 1	
Number of hospitalizations due to acute exacerbation in the past year	0 = 1	1 time = 2	≥ 2 times = 3
Received PR training under the guidance of medical staff	No = 0	Yes = 1	

**Table 4:** Multiple linear regression analysis of factors associated with KAP related to pulmonary rehabilitation among older adults with COPD.

Variables	Partial regression coefficient	Standard error	Standardized regression coefficient	*T-*value	*p-*value	95.0% Confidence interval	
						Lower limit	Upper limit
Constant	73.727	6.967	—	10.583	0.001	59.957	87.497
Hospital visited	−6.064	2.657	−0.151	−2.282	0.024	−11.315	−0.812
Age	−3.850	1.022	−0.253	−3.766	0.001	−5.871	−1.829
Is there a primary caregiver	9.994	2.775	0.240	3.601	0.001	4.509	15.479
Number of hospitalizations due to acute exacerbation in the past year	3.150	1.901	0.113	1.656	0.100	−0.609	6.908
Received PR training under the guidance of medical staff	15.728	2.645	0.398	5.947	0.001	10.500	20.955

R^2^ = 0.377, adjusted R^2^ = 0.355, F = 17.402, *p* < 0.001.

## 4 Discussion

The PR KAP score among older adults with COPD in the medical community was 68.63 ± 19.23, corresponding to a score rate of 52.79%, which reflects a medium-to-low level and is lower than the PR-related KAP score reported among patients with acute exacerbations of COPD in another study (9).

Although PR is recognized as a key therapeutic component in COPD management and its clinical benefits have been confirmed by established guidelines, participation in PR remains limited both nationally and internationally, and overall awareness of PR among patients with COPD is insufficient (10).

In the present study, the mean score for the knowledge dimension was 32.57 ± 11.79, with a score rate of 54.29%, indicating inadequate knowledge of PR among older adults with COPD. This limited knowledge may be attributed to multiple factors, including age-related differences in health awareness and restricted access to relevant information. Older adults primarily relied on guidance from medical staff to obtain PR-related knowledge. However, within the medical community, healthcare professionals may themselves have insufficient understanding of PR. Although PR is widely acknowledged as a core component of COPD management, personnel in primary healthcare institutions may lack systematic training and practical experience in PR, which limits their ability to effectively convey its importance and implementation strategies to patients contributing to poor patient awareness. This is reflected in our finding that 62% of participants reported not having received professional rehabilitation guidance from medical staff, consistent with previous evidence that healthcare personnel may have limited knowledge related to PR, which may reduce the frequency of rehabilitation guidance provided during routine clinical care (11, 12). These findings indicate a clear need to strengthen professional education and training related to PR among medical personnel within the medical community. The medical community model offers a practical framework for optimizing medical resource allocation and enhancing service capacity in primary healthcare institutions. Through strengthened coordination between higher-level and lower-level institutions, county-level hospitals can reinforce internal training, provide targeted technical support to primary healthcare personnel, and simultaneously enhance patient education to improve awareness of PR and support its effective implementation.

Older adults with COPD in the medical community demonstrated generally positive attitudes toward PR. The attitude dimension score was 15.77 ± 3.77, corresponding to a score rate of 78.87%, indicating favorable acceptance of PR. This finding is broadly consistent with the results reported in a study on patients with COPD in a tertiary hospital setting (13). Despite limited knowledge, study participants maintained relatively optimistic attitudes toward PR, which may reflect a strong desire for health improvement and trust in healthcare providers. Even in the absence of sufficient understanding, patients may remain willing to attempt PR. During PR implementation, dissemination of PR-related knowledge should be strengthened while continuing to leverage these positive attitudes to enhance participation and acceptance.

The practice dimension score was 20.28 ± 8.50, with a score rate of 40.56%, indicating low adherence to PR behavior among older adults with COPD. According to the KAP framework, inadequate knowledge and cognitive limitations related to PR may hinder the development and maintenance of appropriate health behaviors. In addition, physical limitations, insufficient professional guidance, and a lack of supervision during the rehabilitation process may contribute to discontinuation of PR programs.

As leading institutions within the medical community, county-level hospitals should assume a guiding role by promoting knowledge sharing and skills transfer and by collaboratively advancing the dissemination and implementation of PR across the medical community.

PR-related KAP among older adults with COPD in the medical community was influenced by multiple factors. The findings indicated that the medical institution visited, age, presence of a primary caregiver, and guidance from medical staff were significant factors associated with PR-related KAP among older adults with COPD.

1. Medical institution visited: Participants who received care at county-level hospitals demonstrated higher PR-related KAP levels than those who visited primary healthcare institutions. This difference may be attributable to the relatively richer medical resources available at county hospitals, as well as higher disease awareness among individuals seeking care at these facilities. Such individuals may be more proactive in seeking medical information and services, thereby enhancing their acceptance and understanding of PR. Primary healthcare institutions play a central role in the functioning of the county medical community, and substantial potential remains for improving PR-related KAP among patients with COPD at this level (14). A systematic assessment of PR needs and existing gaps across regions and institutions, strengthening of weaker implementation components, optimization of resource allocation, and enhancement of implementation efficiency are warranted to improve the overall quality of PR delivery within the medical community.
2. Age: Younger participants demonstrated higher PR-related KAP levels, which is consistent with findings reported in a previous study (15). With increasing age, older adults often experience declines in cardiopulmonary function, physical capacity, and memory, which may directly affect PR effectiveness and limit the ability to comprehend and apply PR-related knowledge. In addition, exercise intolerance in some individuals may prevent completion of PR activities, thereby negatively influencing KAP levels. To effectively improve PR-related KAP, early-stage education and intervention during the initial phases of COPD identification and management in the medical community are recommended.
3. Primary caregiver: Participants with a designated primary caregiver demonstrated higher PR-related KAP levels. As the home environment plays a critical role in the daily lives of individuals with COPD, support from caregivers, including emotional encouragement, daily care, and assistance with rehabilitation activities, can greatly influence patient motivation and adherence to PR. Inadequate family support has been reported to adversely affect health outcomes in individuals with COPD (16). Evidence indicates that hospital-community-family treatment alliances can improve adherence to PR among individuals with chronic respiratory diseases (17). Under the guidance of county-level hospitals and primary healthcare institutions, promoting the involvement of family caregivers in home-based PR may represent an effective strategy for improving rehabilitation outcomes (18).
4. Guidance from medical personnel: Rehabilitation outcomes are closely linked to professional guidance from healthcare providers. Participants who received professional guidance from medical staff demonstrated higher PR-related KAP scores. Within the medical community, integration and sharing of medical resources across institutions facilitate the provision of more comprehensive and continuous PR services. Although PR delivery within the medical community represents an effective approach, some patients may not be promptly referred to primary healthcare institutions for continued PR following treatment at higher-level hospitals. Referral rates for PR among patients with COPD remain low (19, 20). Standardized COPD screening, timely referral, and comprehensive rehabilitation management for patients experiencing acute exacerbations at the primary healthcare level may support improved continuity of care (21). Preventive strategies proposed in previous studies may also inform PR implementation for patients with COPD (22). Leveraging the complementary strengths of institutions at different levels within the medical community is recommended, with primary healthcare institutions responsible for daily rehabilitation management and follow-up and county-level hospitals providing professional technical support. Through the joint establishment of PR teams, integration of rehabilitation into medical community policies, strengthened communication and information sharing, and implementation of hierarchical diagnosis, treatment, and referral systems, patients may receive continuous and effective PR services within the medical community.

## 5 Limitations

This study has several limitations that should be considered when interpreting the findings. First, this was a cross-sectional study; therefore, we can only describe the current status of KAP regarding pulmonary rehabilitation among older adults with COPD within the medical community, with no ability to infer causality. Second, although patients were recruited from multiple healthcare institutions (one tertiary hospital and three primary healthcare centers), all participants were from a single county medical community in Zhejiang Province. This may limit the generalizability of our findings to other regions with different healthcare organization models, socioeconomic contexts, or patient populations. Third, the use of convenience sampling and self-reported questionnaires may introduce selection and recall bias. In addition, the sample size for subgroup analyses (e.g., different age groups or medical institutions) was relatively limited, which may have restricted our ability to detect additional significant associations. Despite these limitations, our sample size met the statistical requirements, and the findings provide valuable insights for optimizing pulmonary rehabilitation services within the medical community model. Future multicenter studies with longitudinal designs and more diverse populations are warranted to validate and extend our findings.

## 6 Conclusion

In summary, the overall level of knowledge, attitude, and practice related to pulmonary rehabilitation among older adults with COPD in the medical community remained relatively low, representing a key barrier to the effective implementation and expansion of pulmonary rehabilitation. Strengthened integration of prevention and treatment services between county-level hospitals and primary healthcare institutions within the medical community is warranted to promote closer and more effective collaboration. Optimizing the rational allocation and efficient utilization of medical resources across the medical community may contribute to meaningful improvements in the overall quality and effectiveness of COPD management.

## Data Availability

The original contributions presented in the study are included in the article/supplementary material, further inquiries can be directed to the corresponding author.
